# Supplementary material for: Patterns of Intron Gain and Loss in Fungi
Source: PLoS Biol. 2004 Nov 30;2(12):e422. doi: 10.1371/journal.pbio.0020422 (PMC532390; doi:10.1371/journal.pbio.0020422)
Supplement: Table S1 — Also available at http://genes.mit.edu/NielsenEtAl/. (4.3 MB ZIP). [file pbio.0020422.st001.zip › NielsenEtAl/html/895.html]

AN7632.1.NCU06652.1.MG06011.1.FG10200.1


```
 CLUSTAL W (1.82) Multiple Sequence Alignments - Introns Inserted


Sequence 1: MG06011.1	389 aa
Sequence 2: FG10200.1	384 aa
Sequence 3: AN7632.1	379 aa
Sequence 4: NCU06652.1	380 aa
Alignment Length: 394 aa
Number Identitical Residues: 280 aa
Alignment Score (without introns) 12216


MG06011.1 	-MSTVGK~VSKANTRM0TITCK~AAVAWEAGKDLSIEDIEVAPPKAHEVRIEIYHTGVCH
NCU06652.1	MASTVGK0T-------~-ITCK~AAIAWGAGQELSYEDVEVAPPKAHEVRIQIKHTGVCH
FG10200.1 	-MSTAGK0T-------~-ITCK~AAVAWEAGKPLSIEDIEVAPPKANEVRIQIYYTGVCH
AN7632.1  	MASTVGK0T-------~-ITCK0AAIAWAAGEPLSIEDVEVAPPKAHEVRIQVLHTGVCH
          	  **.** .         **** **:** **: ** **:*******:****:: :*****

MG06011.1 	TD~AYTLSGKDPEGAFPIVLGHEGAGIVESVGEGVTNVKVGDHVVALY2TPECKECKFCK
NCU06652.1	TD1AYTLSGKDPEGAFPVILGHEGAGIVESVGEGVTNVKPGDHVIALY~TPECKECKFCK
FG10200.1 	TD1AYTLSGKDPEGAFPIVLGHEGAGIVESIGEGVTNVKVGDHVVALY2TPECKECKFCK
AN7632.1  	TD1AYTLSGKDPEGAFPVILGHEGAGIVESVGEGVTSVKPGDHVIALY2TPECRECKFCK
          	** **************::***********:*****.** ****:*** ****:******

MG06011.1 	SGKTNLCGKIRATQGKGLMPDGTSRFKCKGKDLLHFMGTSTFSQFTVVADISVVAVQPEA
NCU06652.1	SGKTNLCGKIRATQGRGVMPDGTSRFRARGQDILHFMGTSTFSQYTVVADISVVAVNPEA
FG10200.1 	SGKTNLCGKIRATQGKGLMPDGTSRFKCKGKDLLHFMGTSTFSQYTVVADISVVAIEHDA
AN7632.1  	SGKTNLCGKIRATQGKGVMPDGTSRFKARGKDLLHFMGTSTFSQYTVVADISVVAVTDKI
          	***************:*:********:.:*:*:***********:**********:  . 

MG06011.1 	PMDRTCLLGCGITTGYGAARVTANVEEGSSLAVFGAGCVGLSVVQGAVINKAGKIIVVDV
NCU06652.1	PMDRTCLLGCGITTGYGAATITANVEKGSTVAIFGAGCVGLSVIQGAVANGASKIIAVDV
FG10200.1 	PMDRTCLLGCGITTGYGAATITANVQAGDNVAIFGAGCVGLSVVQGAAARKAGKIIVVDV
AN7632.1  	PTDRSCLLGCGITTGYGAAVVTAKVEEGSNIAVFGAGCVGLSVMQGAVKNKAGKIIAVDV
          	* **:************** :**:*: *..:*:**********:***. . *.***.***

MG06011.1 	NPAKEEWARKFGATDFVNPTKLPEGKTVVDALVELTDGGCDYTFDCTGNVQVMRAALEAC
NCU06652.1	NPSKEEWSRKFGATDFVNPSTLPEGQSVVDKLIELTDGGCDYTFDCTGNVKVMRAALEAC
FG10200.1 	NPSKKEWGEKFGATDFVNPTELGD-QSIQDKLIEMTDGGCDYTFDCTGNVGVMRAALEAC
AN7632.1  	NDAKEEWSRKFGATDFVNPTKLN-GQTIQEKLIEMTDGGCDYTFDCTGNVGVMRAALEAC
          	* :*:**..**********: *  .::: : *:*:*************** *********

MG06011.1 	HKGWGESIIIGVAAAGQEISTRP~FQLVTGRVWKGCAFGGIKGRSQLPGLVDDYLQGRLK
NCU06652.1	HKGWGQSIIIGVAAAGQEISTRP1FMLVTGRVWRGSAFGGVKGRSQLPGLVEDYLNGKIK
FG10200.1 	HKGWGESIVIGVAAAGQEISTRP1FQLVTGRVWRGCAFGGVKGRSQLNDLVADYKKGDLK
AN7632.1  	HKGWGESIVIGVAAAGQEISTRP1FQLVTGRVWRGCAFGGIKGRTQLPGLVDDYLDGKLK
          	*****:**:************** * *******:*.****:***:** .** ** .* :*

MG06011.1 	VDEFITHRKKLVEINNAFETMKQGDCIRAVVDMRAV----
NCU06652.1	VDELITHRKKLAEINNAFEVMHQGDCVRAVVDMS------
FG10200.1 	VDEFITHRKTLGEINNAFDTMKQGDCIRAVVDMQKLEPKI
AN7632.1  	VDEFITHREPLANINTAFEQMKAGDCIRCVVDLS------
          	***:****: * :**.**: *: ***:*.***:
```
